# Faricimab treat-and-extend approach for neovascular age-related macular degeneration: insights from real-world clinical practice

**DOI:** 10.1186/s40942-025-00776-0

**Published:** 2025-12-05

**Authors:** Jorge Ruiz-Medrano, Iulia Pana, María García-Zamora, Ignacio Flores-Moreno, Mariluz Puertas, José Mª Ruiz-Moreno

**Affiliations:** 1 Department of Ophthalmology, Puerta de Hierro-Majadahonda University Hospital, C/ Manuel de Falla, 1, Majadahonda, Madrid, 28222 Spain; 2 Ocular Microsurgery Institute IMO, Madrid, Spain; 3 Department of Ophthalmology, Castilla-La Mancha University, Albacete, Spain

**Keywords:** Neovascular-AMD, Vascular endothelial growth factor inhibitors, Faricimab, Treat-and extend, Treatment interval

## Abstract

**Purpose:**

To evaluate the clinical outcomes of the switch to faricimab in a treat-and-extend (T&E) regimen patients with neovascular age-related macular degeneration (nAMD).

**Methods:**

This prospective cohort study included consecutive patients with nAMD who had previously been treated with anti-VEGF agents in a T&E regimen, with treatment intervals (TI) that could not be extended beyond 12 weeks, and a minimum follow-up of 24 weeks. These patients were switched to faricimab therapy in a T&E regimen for at least 6 months. The primary endpoint was the TI between intravitreal injections (IVIs), and the secondary endpoint was the mean change in best-corrected visual acuity (BCVA) from baseline to the last follow-up visit (LFUV).

**Results:**

A total of 225 eyes from 188 patients were included, with a mean age of 79.6 ± 7.4 years. Previous anti-VEGF treatments included ranibizumab (*n* = 34), aflibercept (*n* = 144), brolucizumab (*n* = 6), and bevacizumab (*n* = 41). TI1 (5.9 ± 2.0 weeks) matched the prior treatment interval. Significant increases in treatment intervals were observed at subsequent time points (TI2: 8.2 ± 3.2 weeks, TI3: 10.1 ± 3.9 weeks, TI4: 10.7 ± 4.3 weeks, TI5: 9.9 ± 4.0 weeks, and TI6: 8.5 ± 4.4 weeks; *p* < 0.001). BCVA remained stable, going from 0.41 ± 0.23 to 0.43 ± 0.24 (*p* = 0.0112). The mean number of injections was 5.9 ± 1.9, with a mean follow-up duration of 51.4 ± 11.8 weeks.

**Conclusions:**

The switch to faricimab in a T&E regimen significantly increased treatment intervals maintaining BCVA in patients with nAMD under other anti-VEGF treatments. No serious adverse events were reported. Longer follow-up is needed to confirm these results.

**Supplementary Information:**

The online version contains supplementary material available at 10.1186/s40942-025-00776-0.

## Highlights

Results from clinical trials have shown the safety and efficacy of faricimab for AMD patients

Real-life patient often differ from those from clinical trials so results can seldom be extrapolated

This study provides results of faricimab in pretreated neovascular AMD patients in real-world clinical practice

Different factors (age, initial BCVA and number of previous intravitreal injections) may play a role on the efficacy of the drug in refractory cases

**Supplementary Information:**

The online version contains supplementary material available at 10.1186/s40942-025-00776-0.

## Introduction

Age-related macular degeneration (AMD), including both its neovascular (nAMD) and non-neovascular forms, is a leading cause of vision loss in the elderly population [1]. The treatment of nAMD typically involves intravitreal (IV) injections of vascular endothelial growth factor inhibitor (anti-VEGF) drugs, which have significantly enhanced visual outcomes for these patients [2]. However, intravitreal therapy places a substantial burden on patients, healthcare providers, and the public health system.

The response to anti-VEGF therapy is variable, with some patients exhibiting resistance, relapses, or lack of response to treatment [3]. Approximately 30% of patients are considered non-responders [4, 5]. In this context, the inhibition of angiopoietin-2 (Ang-2) has been proposed as a potential therapeutic strategy. The approval of faricimab, a dual-acting agent that inhibits both vascular endothelial growth factor (VEGF)-A and Ang-2, has introduced new treatment options for patients with nAMD [6, 7].

The pivotal phase 3 studies, TENAYA and LUCERNE, demonstrated the effectiveness of faricimab in treating naïve nAMD [8–10]. By inhibiting two key pathways involved in macular neovascularization, these trials observed an increase in the interval between treatment injections [8]. However, its efficacy and therapeutic potential in patient’s refractory to conventional anti-VEGFs in real-world clinical practice remain not fully defined.

This study aimed to evaluate the ability of faricimab to extend treatment intervals (TI) in a “Treat and Extend” (T&E) regimen for patients with nAMD under other anti-VEGF agents (i.e., ranibizumab, aflibercept, brolucizumab, and bevacizumab).

## Methods

The study was approved by the Ethics Committee of Puerta de Hierro-Majadahonda University Hospital (protocol number 31/25) and conducted in compliance with the principles of the Declaration of Helsinki, International Council for Harmonization (ICH) guidelines, Good Clinical Practice (GCP) standards, and applicable Spanish laws.

To ensure patient confidentiality, all identifying data were either encrypted or appropriately anonymized.

### Study participants

This prospective cohort study enrolled consecutive male and female patients aged ≥ 18 years with neovascular age-related macular degeneration (nAMD) undergoing treatment with anti-VEGF agents in a treat-and-extend (T&E) regimen, whose extension intervals could not be prolonged beyond 12 weeks while maintaining a dry retina, and who had a minimum follow-up period of 24 weeks. Patients were offered a switch in therapy to explore the possibility of further interval extension; those who consented were transitioned to faricimab within a T&E protocol for at least 6 months. When both eyes fulfilled the inclusion criteria, both were incorporated into the analysis. The study protocol specified that treatment intervals be extended or shortened in 4-week increments based on anatomical findings, with extension permitted only when complete fluid resolution was observed.

All patients underwent a complete ophthalmological examination, which included decimal best corrected visual acuity (BCVA), slit-lamp examination of the anterior segment, intraocular pressure measurement using Goldmann tonometry, indirect fundus ophthalmoscopy, and multimodal imaging (fundus color photography and spectral domain optical coherence tomography (SD-OCT) using Heidelberg Spectralis or Topcon SS Triton. These examinations were performed on both eyes if they met the inclusion criteria.

### Treat and extend regimen

After the approval of faricimab in Spain, this protocol was implemented in our clinical practice in nAMD patients under treatment with other anti-VEGF agents and treatment interval (TI) of < 12 weeks. By protocol design, the initial treatment interval (TI1) corresponded to the last interval prior to switching to faricimab. In subsequent visits, if the retina was dry (no sub- or intraretinal fluid), the treatment interval was extended by 4 weeks, with intravitreal injections (IVIs) of faricimab administered according to the T&E regimen. If fluid was identified on OCT images, TI were shortened accordingly.

### Study outcomes

The primary endpoint was the TI between faricimab IVIs. The secondary endpoint was the mean change in BCVA from baseline (initial interval) to the last follow-up visit (LFUV). Baseline characteristics were studied in order to establish their potential influence in patients’ response to the switch of treatment including: type of CNV, accumulated number of previous IVIs, previous anti-VEGF used, initial BCVA, baseline TI, age, gender and laterality.

“Optimal responders” were defined as those whose TI was extended by ≥8 weeks. “Non optimal responders” did not reach an extension of 8 weeks.

### Statistical analysis

Statistical analyses were conducted using IBM SPSS Statistics Software (version 29.0.2.0, Chicago, IL, USA). Continuous variables were expressed as mean ± standard deviation (SD), while categorical variables were presented as frequencies and percentages. The Kolmogorov-Smirnov test was applied to assess the normality of variable distributions. Paired Student’s t-test was used to compare differences between TIs and BCVA. Student t test was used to compare quantitative variables between optimal and non-optimal responders.

Categorical variables were compared using chi-square test. A p-value of less than 0.05 was considered statistically significant.

## Results

Of the 245 eyes that met the inclusion and exclusion criteria, 20 were excluded from the final analysis for various reasons: 1 eye due to retinal detachment, 5 eyes from 3 patients who died, 7 eyes from 5 patients who discontinued treatment at their request due to inability to attend follow-up visits, 1 eye with massive subretinal hemorrhage, 2 eyes from a patient who changed hospitals due to relocation, and 4 eyes from 2 patients who voluntarily withdrew from the study.

A total of 225 eyes of 188 subjects were analyzed. The mean age of the participants was 79.6 ± 7.4 years, with a range from 58 to 96 years. Among the 188 patients, 122 (54.2%) were women, and 123 (54.9%) had their left eye included in the study. Prior to enrollment, 34 patients had been treated with ranibizumab, 144 with aflibercept, 6 with brolucizumab, and 41 with bevacizumab. No significant safety incidents were observed (Table 1).

**Table 1:** Baseline demographics

Baseline characteristics	Treatment-experienced patients (*N* = 245)
Mean ± SD age, years (range)	79.9 ± 7.3 (58–95)
Sex, %	
Female patients	53.3
Mean ± SD BCVA pre-switch, (range)	0.41 ± 0.59 (0.1–0.98)
Mean ± SD treatment interval pre-switch, weeks (range)	6 ± 1.97 (4–10)
Mean ± SD number of injections pre-switch, n	18.8 ± 14.78
Breakdown of prior treatment pre-switch, %	
Switched from aflibercept	65.6
Switched from ranibizumab reference product or biosimilar	15.8
Switched from brolucizumab	1.6
Switched from bevacizumab	17.0
Mean ± SD length of follow-up post-switch, weeks (range)	32.75 ± 9.62 (6–60)

BCVA: best-corrected visual acuity; SD: Standard deviation

### Treatment intervals

By design, the initial treatment interval (TI1) was set at 5.9 ± 2.0 weeks (range 4–10 weeks), which corresponded to the TI prior to switching to faricimab. After switching to faricimab, significant increases in the TI were observed at each subsequent time point compared to TI1 (*p* < 0.0001 for each, respectively). These differences remained significant in subsequent intervals (Table 2).

**Table 2:** Overview of the treatment intervals (TI) throughout the study follow-up

Interval	*N*	Mean ± SD (weeks)	Difference from IT1(Student t test)		
			Mean ± SD	95%CI	*p*
TI1	225	5.9±2.0	NA		
TI2	225	8.2 ± 3.2	2.3 ± 2.7	1.8 to 2.8	< 0.0001
TI3	224	10.1 ± 3.9	4.2 ± 3.1	3.6 to 4.8	< 0.0001
TI4	212	10.7 ± 4.3	4.8 ± 3.3	4.2 to 5.4	< 0.0001
TI5	169	9.9 ± 4.0	4.0 ± 3.0	3.4 to 4.6	< 0.0001
TI6	117	8.5 ± 4.4	2.6 ± 3.0	1.9 to 3.3	< 0.0001
LFUV	225	11.6±24.3	5.7 ± 17.2	2.5 to 8.9	0.0005

N: Number of eyes; SD: Standard deviation; CI: Confidence interval; TI: Treatment interval; LFUV: Last follow-up visit

The comparison between the initial mean interval (TI1) and the final recorded mean interval (last follow-up) showed a statistically significant difference: 11.6 ± 24.3 weeks (range 4–20 weeks, *n* = 225; *p* < 0.001, paired Student’s t-test). Treatment intervals could only be extended if the anatomical response was deemed complete, with complete absence of intra- or subretinal fluid. These figures indicate a degree of heterogeneity of the data has to be taken into account in order to potentially extrapolate these results.

To give the data more meaningful value, we have performed an analysis of a subgroup with more than 52 weeks of follow-up (125 eyes, with a mean follow-up of 59.6 ± 7.7; from 52 to 88 weeks). TI increases from 6.4 ± 1.9 to last TI of 11.0 ± 3.9 (*p* < 0.001; paired Student’s t-test), whilst BCVA varies from to 0.44 ± 0.23 to 0.45 ± 0.24 respectively (*p* = 0.08; paired Student’s t-test).

**Fig. 1:**
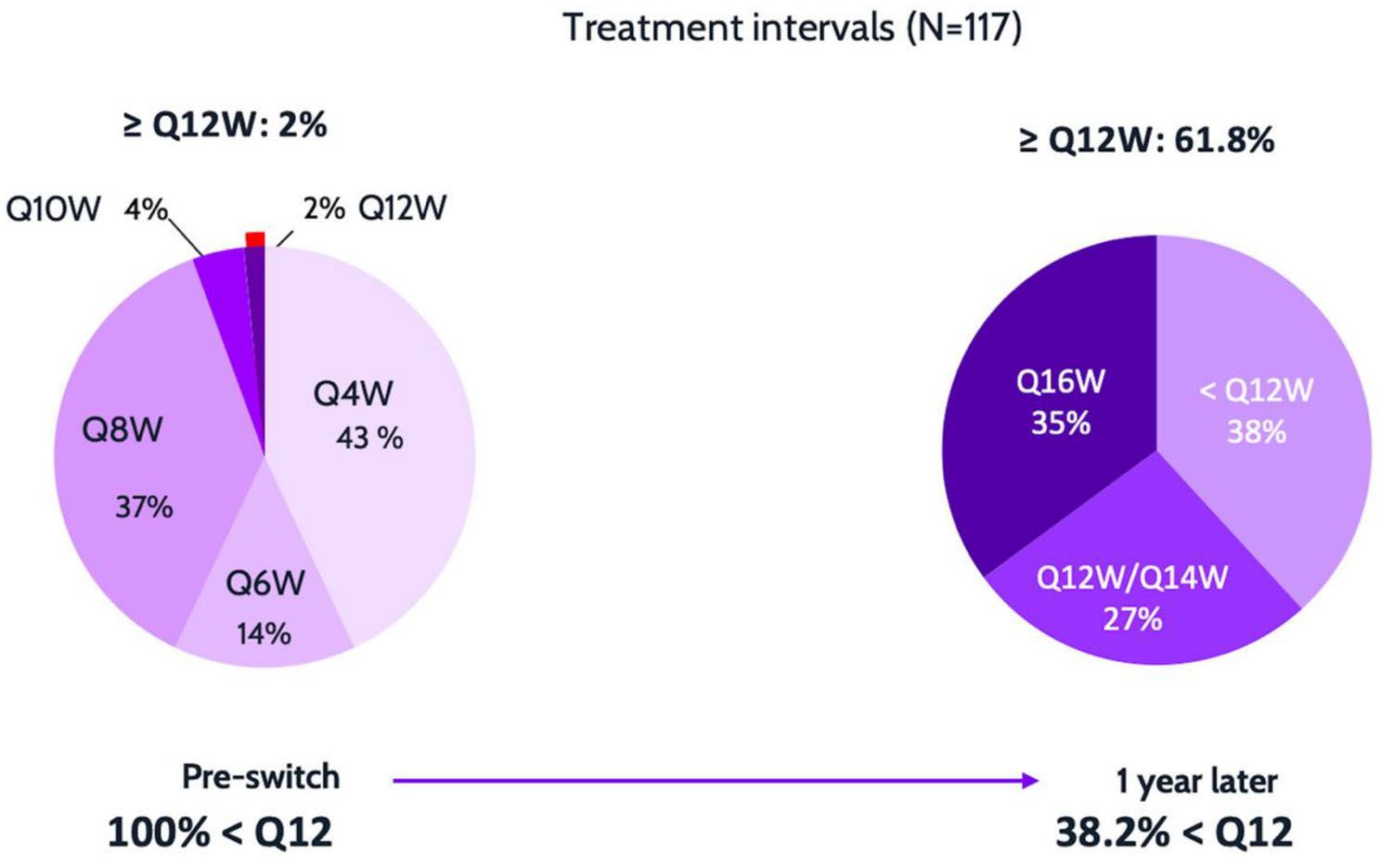
Comparison of patient proportions based on treatment intervals (TI) with various vascular endothelial growth factor inhibitors (initial interval) and at the last follow-up visit after switching to intravitreal faricimab. Statistically significant differences were observed (*p* < 0.0001, Chi-squared test)

Regarding the frequency distribution of treatment Intervals, the initial distribution showed 225 eyes (100%) with treatment intervals of less than 12 weeks. At the last follow-up, 86 eyes (38.2%) remained with treatment intervals of less than 12 weeks, while 139 eyes (61.8%) had intervals of > 12 weeks, with 79 eyes (35.1%) having intervals of > 16 weeks, and 9 eyes (4%) having intervals of 20 weeks (*p* < 0.0001, Chi-squared test) (Fig. 1).

Of the 225 eyes, 8 (3.5%) had a shorter treatment interval at the last follow-up compared to the initial interval. In 28 eyes (12.4%), the treatment interval could not be extended and remained the same. In the remaining 189 eyes (84%), the treatment interval was successfully lengthened, indicating that the objective was achieved in 84% of cases.

The mean number of IVIs administered was 5.9 ± 1.9 (range: 2–12), with a mean follow-up time of 51.4 ± 11.8 weeks (range: 24–88 weeks).

### Best corrected visual acuity

**Fig. 2:**
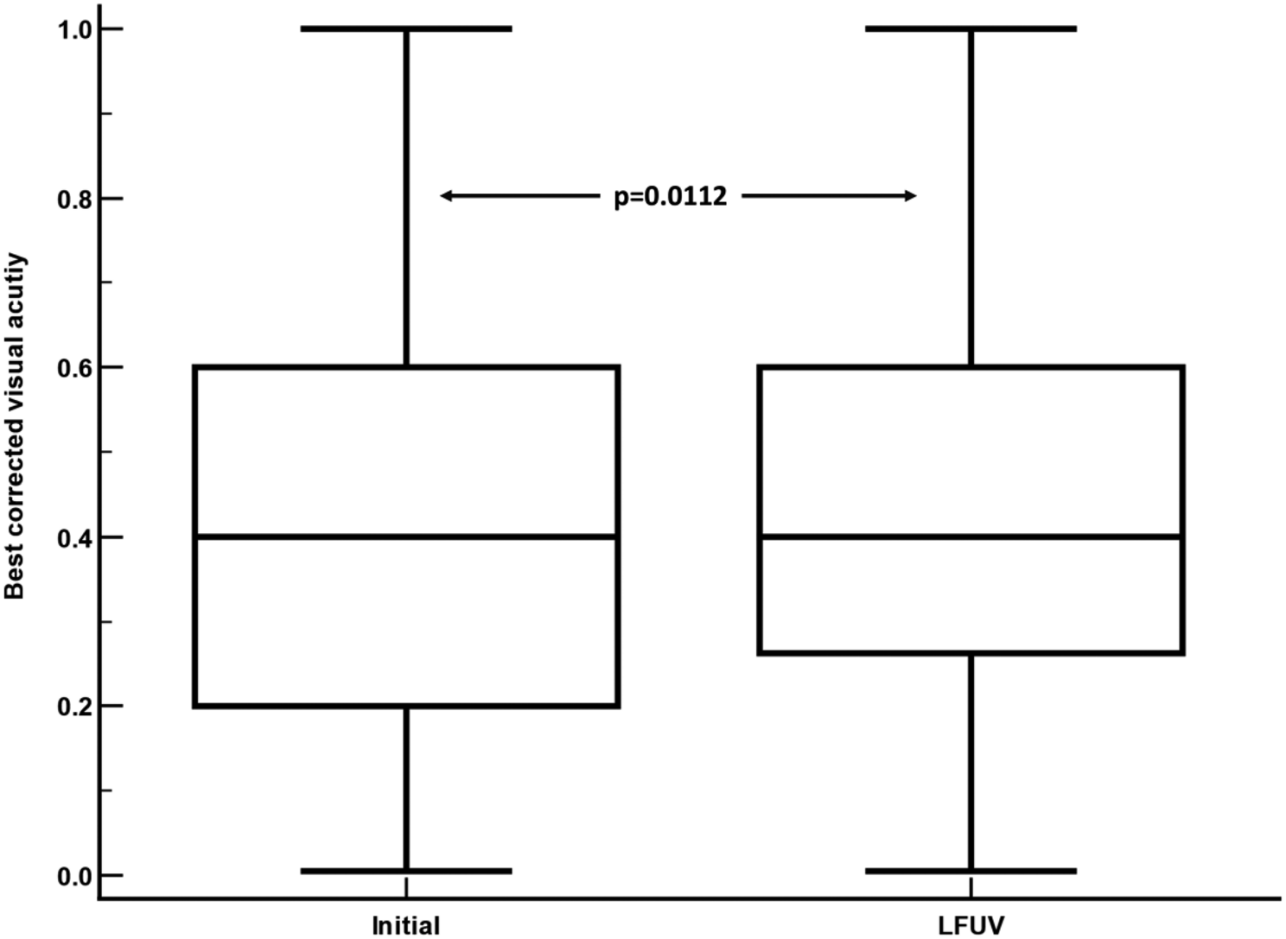
A comparison of the best corrected visual acuity (BCVA) at initial interval and at the last follow-up visit (LFUV). BCVA improved significantly after switching to intravitreal faricimab injections. mean difference from initial interval: +0.015; 95% confidence interval: 0.003 to 0.026; *p* = 0.0112. Paired samples two-way Student t test)

BCVA showed remained stable, increasing from 0.41 ± 0.59 at baseline (prior to switching to faricimab) to 0.43 ± 0.58 at the LFUV (Fig. 2). The mean difference was + 0.015 (95% confidence interval: 0.003 to 0.026; *p* = 0.0112, paired two-tailed Student’s *t*-test).

123 eyes were considered “non optimal responders” and 103 were labeled as “optimal responders”. No statistically significant differences were found when comparing these two groups regarding laterality, gender, type of CNV, pre-switch treatment or baseline TI. On the other hand, statistically significant differences were obtained when comparing age, number of previous IIV and initial BCVA (see Table 3).

**Table 3:** Quantitative variables comparison between “optimal responders” and “non optimal responders”

	Group	*n*	Mean	SD	*P* value*
Age	NOR	123	80.37	7.33	0.04
	OR	102	78.67	7.35	
Initial TI	NOR	123	6.08	2.07	0.18
	OR	102	5.84	1.92	
Initial BCVA	NOR	123	0.39	0.21	0.01
	OR	102	0.46	0.25	
Number IVIs	NOR	123	21.30	16.53	0.003
	OR	102	15.78	11.73	

TI: Treatment interval; BCVA: Best Corrected Visual Acuity; IVI: Intravitreal injections; NOR: Non optimal responders; OR: Optimal responders. *: Student t test

## Discussion

Extending TIs between IVIs in nAMD is a key priority. The treatment burden and the limited durability of currently available anti-VEGF agents have been identified as the most significant unmet needs in nAMD management, as reported by the 2020 EURETINA Clinical Survey Outcomes [11].

Faricimab, as previously discussed, exhibits a dual mechanism of action that has been shown to extend TIs in in nAMD treatment-naïve patients [6–9, 12]. Recent findings from real-world clinical practice further support its efficacy and durability. Stanga et al. reported short-term real-world outcomes in a cohort of nine patients, both naïve and non-naïve, with nAMD, demonstrating the therapeutic efficacy and prolonged durability of faricimab [5].

Tamiya et al. evaluated the efficacy of faricimab in 25 patients with nAMD refractory to conventional anti-VEGF therapies. Their study revealed that 56% of treated eyes experienced a reduction or complete absorption of retinal fluid, highlighting its potential in treatment-resistant cases [13]. Similarly, Mut et al. investigated the outcomes of faricimab in six naïve patients and 57 individuals with nAMD who transitioned to faricimab from other treatments. Notably, complete resolution of subretinal and intraretinal fluid was achieved in 40% of the switched patients after four weeks. The authors concluded that faricimab injections demonstrated a high safety profile and produced a statistically significant reduction in macular edema in this cohort [14].

Matsumoto et al. recently published a retrospective interventional case series assessing 1-year outcomes of a loading phase followed by maintenance therapy using a T&E regimen with intravitreal faricimab in 40 eyes with nAMD. The study concluded that this approach is generally safe and effective, resulting in improved visual acuity and reduced exudative changes in eyes affected by nAMD [12].

The results of this clinical practice study, which investigated patients with nAMD and suboptimal response to other anti-VEGF agents, are consistent with previously reported results in the literature. Khodor et al. conducted a retrospective analysis of 135 eyes with nAMD, reporting that 22.2% of patients switched to faricimab reverted to their original therapy. Among the remaining 105 eyes, 62.9% were free of fluid at the final follow-up. The authors concluded that faricimab exerts a potent drying effect and demonstrates potential for extending TIs in eyes with persistent fluid despite prior anti-VEGF treatments [15].

Similarly, Ambati et al., in a retrospective study involving 263 eyes from 217 patients, observed that eyes with long-standing nAMD switched to faricimab experienced a significant extension of TIs, maintenance of stable best-corrected visual acuity (BCVA), improvement in central subfoveal thickness CST, and resolution of fluid on OCT in a substantial proportion of patients after one year [16].

Additionally, Goodchild et al., in a single-center retrospective study that evaluated 98 eyes with nAMD, reported that 40% of patients achieved extended TIs, thereby reducing treatment burden [17]. Sim et al. analyzed 130 eyes, demonstrating that patients with nAMD who had experienced a high prior treatment burden maintained BCVA and showed improved anatomical outcomes with extended TIs after switching to faricimab [18].

Similarly, Kataoka et al., through a multicenter retrospective study, evaluated nAMD eyes previously receiving monthly aflibercept injections that were switched to faricimab. Patients initially received faricimab monthly for up to four injections, followed by intervals of at least two months, consistent with the drug labeling. Approximately 40% of eyes successfully extended their TIs from monthly to bimonthly [19].

Qaseem et al. examined the response of 19 refractory nAMD eyes to faricimab, with 47% achieving fluid resolution. The authors concluded that faricimab slightly reduced central subfoveal thickness and decreased fluid in some refractory cases, though its impact on BCVA was minimal [20].

Finally, Cancian et al., in a one-year study of 33 patients previously treated with aflibercept or ranibizumab with suboptimal outcomes, reported significantly prolonged fluid-free TIs and stable BCVA following a switch to faricimab [21].

A new paper on this topic has been published recently by Grimaldi et al. The authors report the efficacy and safety of switching to faricimab in a real-world, retrospective, multicenter, Swiss cohort of patients with pretreated nAMD. 353 eyes were included, with minimun follow-up of 12 months. Mean TI increased from 5.8 ± 2.5 weeks at switch to 8.3 ± 4.2 weeks at 12 months, and extended treatment intervals (≥ 12 week) were achieved in 20% of patients [22].

In another study, Sing et al. [23] evaluated a transition to faricimab in consecutive patients with neovascular age-related macular degeneration (nAMD) who had been receiving 4-week interval treatment with either ranibizumab or 2 mg aflibercept under a treat-and-extend (T&E) regimen. A total of 117 eyes were included. In the 12 months prior to switching, the mean number of injections was 10.1 ± 1.6. Following the switch, the mean treatment interval increased to 6.9 ± 2.3 weeks (*P* < 0.005). At 12 months, 42.9% and 11.4% of patients achieved treatment intervals of ≥ 8 weeks and ≥ 12 weeks, respectively. The authors concluded that, over a 12-month period, patients with nAMD and a history of high treatment burden maintained visual acuity and demonstrated improved anatomical outcomes with extended treatment intervals after converting to faricimab. Given the inherent physician-driven bias in observational designs, the authors recommended conducting a prospective, randomized, controlled trial to confirm these results.

Similarly to the aforementioned studies, the current study represents a large series of eyes switched to faricimab, demonstrating a significant extension in mean TIs between injections (5.7 ± 17.2) and no clinically significant changes in BCVA. A notable shift in TI distribution was observed: the proportion of patients initially treated at intervals shorter than 12 weeks decreased from 100% to 61.8%, with 35.1% achieving intervals of 16 weeks. Overall, 84% of eyes exhibited successful TI extension. These results are comparable or slightly higher that other series to date. The analysis of the subgroup with more than 52 weeks of follow-up reinforces the results of the full study.

According to this analysis the ideal candidates for a switch to faricimab are those with better BCVA, fewer previous IIV and younger age.

Special attention should be paid to the fact thar this study included nAMD patients on other anti-VEGF drugs with a dry retina, but whose TI could not be extended. The switch to faricimab permitted the extension of TIs while maintaining BCVA (which actually showed a non-clinically significant increase). For this reason, retinal thickness, intraretinal fluid, and subretinal fluid have not been analysed.

The primary limitation of this study was the relatively short span of follow-up for patients treated with faricimab and its observational design. In a condition such as nAMD, a 6-month follow-up may be insufficient to fully assess the long-term impact of the drug on disease progression. Although the mean follow-up in our study was nearly one year (51.4 ± 11.8 weeks), the number of eyes decreased after the 7 TIs. The absence of imaging-based endpoints and reliance on morphometric assessment (all patients had a dry retina at the time of switching), as well as potential bias arising from the inclusion of highly compliant, long-treated patients in whom the switch may be more likely to yield favorable outcomes. Nevertheless, these findings are derived from real-world clinical practice, encompassing a substantial number of eyes, and align with previously reported results in the literature. The inclusion of patients with dry retina may pose the question of a potential selection bias but, as per inclusion criteria, none of these patients had been able to maintain anatomical stability if extended beyond a certain interval prior to the switch.

In conclusion, faricimab effectively increases TIs in patients with nAMD managed with a T&E regimen, without compromising visual acuity. Results were better in younger patients with fewer previous treatments and better intial BCVA. Extended follow-up is warranted to validate these findings over a longer timeframe.

## Supplementary Information

Below is the link to the electronic supplementary material.

Supplementary Material 1

## Data Availability

The data that support the findings of this study are not publicly available due to privacy reasons but are available from the corresponding author upon reasonable request.
