# Supplementary figures and images for: Faricimab treat-and-extend approach for neovascular age-related macular degeneration: insights from real-world clinical practice
Source: Int J Retina Vitreous. 2025 Dec 5;12:5. doi: 10.1186/s40942-025-00776-0 (PMC12781570; doi:10.1186/s40942-025-00776-0)

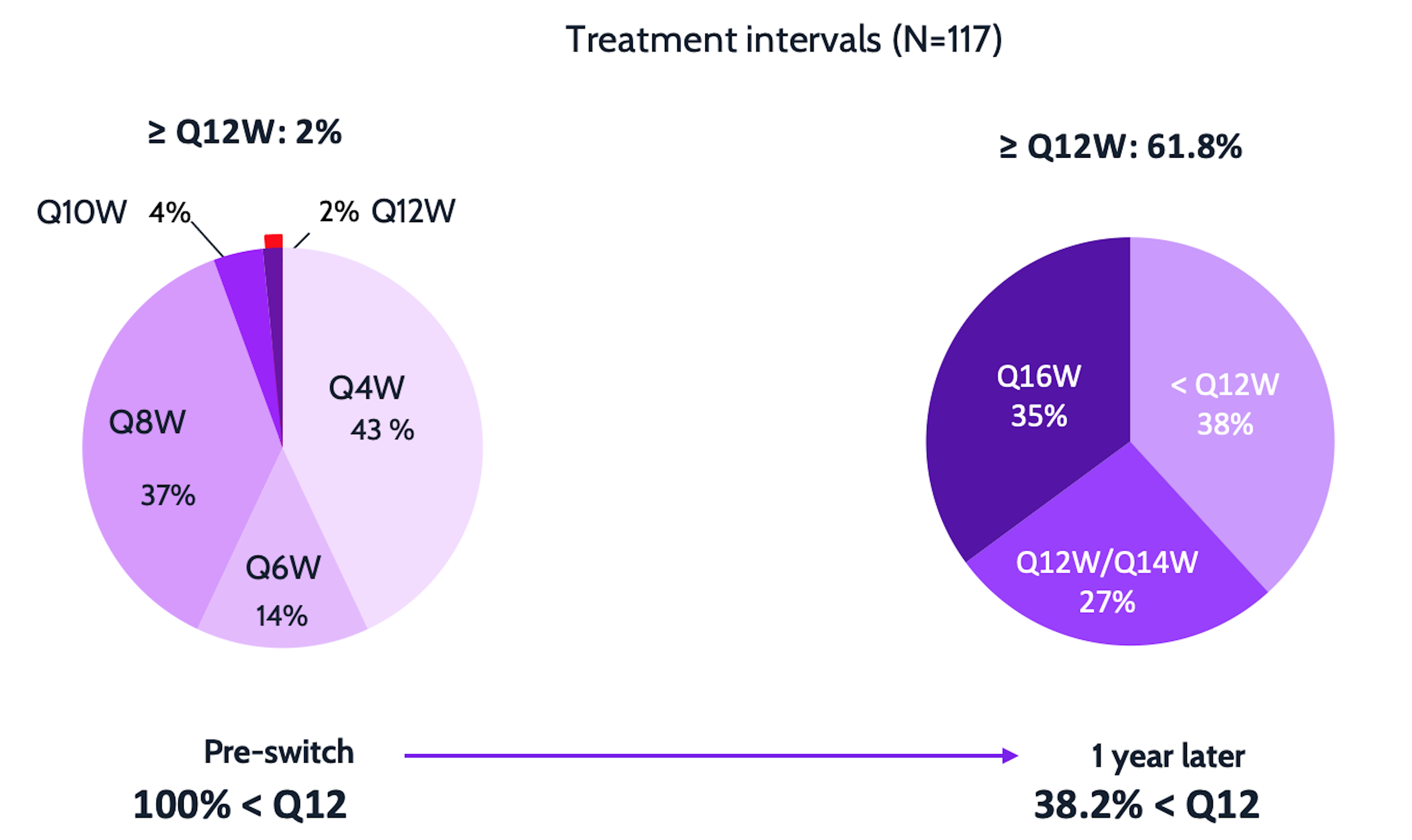

Supplement: Supplementary file 1 — Supplementary Material 1 [file 40942_2025_776_MOESM1_ESM.zip › Figure 1 UD.png]

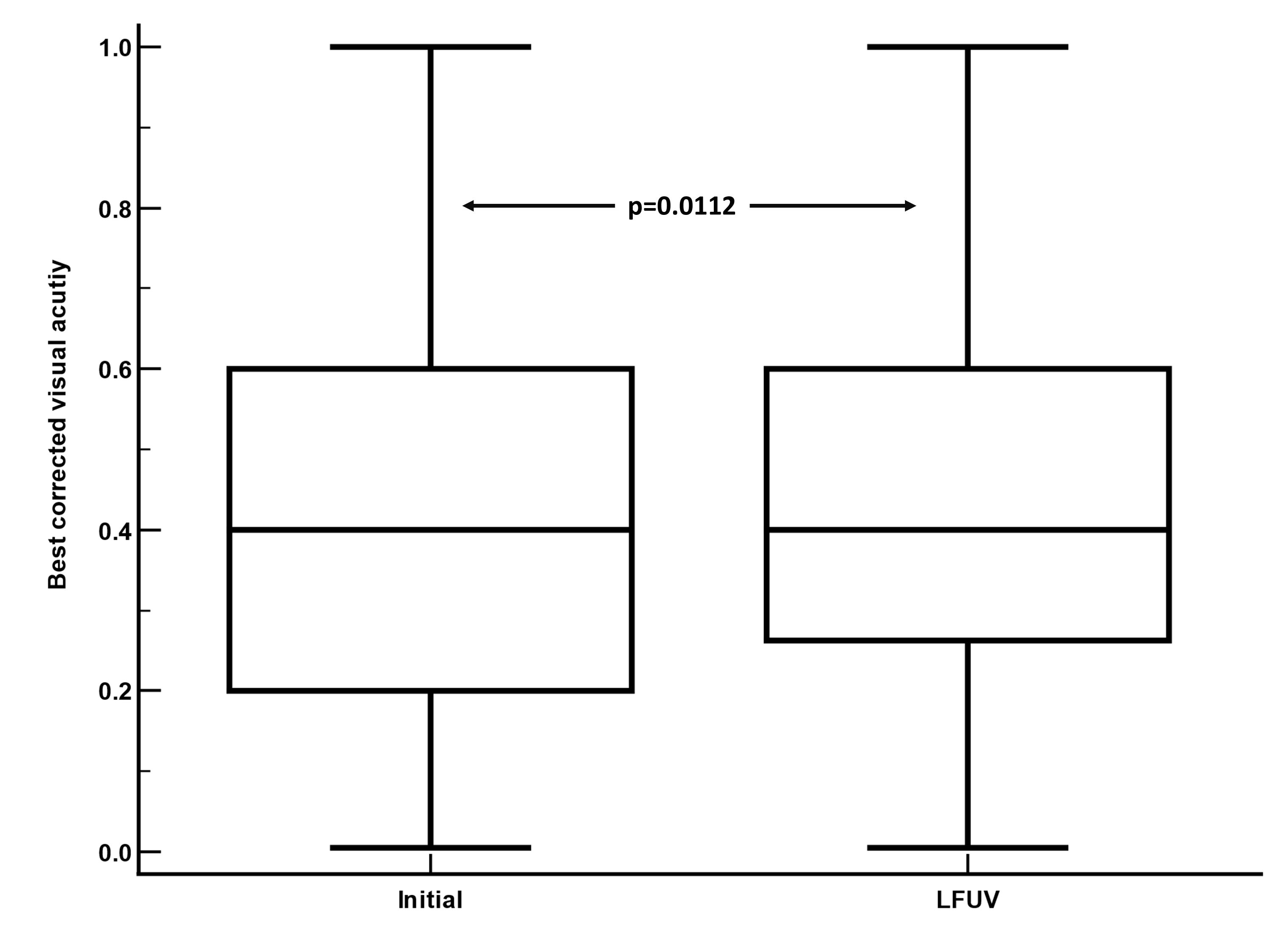

Supplement: Supplementary file 1 — Supplementary Material 1 [file 40942_2025_776_MOESM1_ESM.zip › Figure 2 Faricimab Ruiz-Medrano Ophthalmologica 1.0.TIF]
